# Effectiveness of a Multimodal Community Intervention Program to Prevent Suicide and Suicide Attempts: A Quasi-Experimental Study

**DOI:** 10.1371/journal.pone.0074902

**Published:** 2013-10-09

**Authors:** Yutaka Ono, Akio Sakai, Kotaro Otsuka, Hidenori Uda, Hirofumi Oyama, Naoki Ishizuka, Shuichi Awata, Yasushi Ishida, Hiroto Iwasa, Yuichi Kamei, Yutaka Motohashi, Jun Nakamura, Nobuyuki Nishi, Naoki Watanabe, Toshihiko Yotsumoto, Atsuo Nakagawa, Yuriko Suzuki, Miyuki Tajima, Eriko Tanaka, Hironori Sakai, Naohiro Yonemoto

**Affiliations:** 1 National Center for Cognitive Behavior Therapy and Research, National Center of Neurology and Psychiatry, Kodaira, Tokyo, Japan; 2 Department of Neuropsychiatry, Iwate Medical University, Morioka, Iwate, Japan; 3 Department of Disaster and Community Psychiatry, Iwate Medical University, Morioka, Iwate, Japan; 4 Ijuin Public Health Center, Health, Social Welfare, and Environmental Department, Kagoshima Regional Promotion Bureau, Kagoshima Prefecture, Ijuin, Hioki, Kagoshima, Japan; 5 Department of Social Welfare, Faculty of Health Sciences, Aomori University of Health and Welfare, Hamadate, Aomori, Aomori, Japan; 6 Center for Clinical Sciences, National Center for Global Health and Medicine, Shinjuku, Tokyo, Japan; 7 Research Team for Promoting Independence of the Elderly, Tokyo Metropolitan Institute of Gerontology, Itabashi, Tokyo, Japan; 8 Department of Psychiatry, Faculty of Medicine, University of Miyazaki, Kiyotake, Miyazaki, Japan; 9 Aomori Prefectural Center for Mental Health and Welfare, Sannai, Aomori, Aomori, Japan; 10 Division of Neuropsychiatry, Hirosaki University Graduate School of Medicine, Hirosaki, Aomori, Japan; 11 Department of Laboratory Medicine, National Center Hospital, National Center of Neurology and Psychiatry, Kodaira, Tokyo, Japan; 12 Department of Public Health, Akita University Graduate School of Medicine, Akita, Akita, Japan; 13 Department of Psychiatry, School of Medicine, University of Occupational and Environmental Health, Yahatanishi, Kitakyushu, Fukuoka, Japan; 14 Aira Public Health Center, Health, Social Welfare, and Environmental Department, Aira-Isa Regional Promotion Bureau, Kagoshima Prefecture, Hayato, Kirishima, Kagoshima, Japan; 15 Department of Psychiatry, Asada Hospital, Aki, Hiroshima, Japan; 16 Health Promotion Division, Health and Social Welfare Department, Kagoshima Prefecture, Kagoshima, Kagoshima, Japan; 17 Center for Clinical Research, School of Medicine, Keio University, Shinjyuku, Tokyo, Japan; 18 Department of Adult Mental Health, National Institute of Mental Health, National Center of Neurology and Psychiatry, Kodaira, Tokyo, Japan; 19 The Institute of Humanities and Social Sciences, Nihon University, Setagaya, Tokyo, Japan; 20 Department of Basic Medical Sciences, School of Health Sciences, Graduate School of Medicine, Gunma University, Maebashi, Gunma, Japan; 21 Department of Epidemiology and Biostatistics, Translational Medical Center, National Center of Neurology and Psychiatry, Kodaira, Tokyo, Japan; 22 Department of Neuropsychopharmacology, National Institute of Mental Health, National Center of Neurology and Psychiatry, Kodaira, Tokyo, Japan; 23 NOCOMIT-J Group, National Center of Neurology and Psychiatry, Kodaira, Tokyo, Japan; University of Rochester, United States of America

## Abstract

**Background:**

Multilevel and multimodal interventions have been suggested for suicide prevention. However, few studies have reported the outcomes of such interventions for suicidal behaviours.

**Methods:**

We examined the effectiveness of a community-based multimodal intervention for suicide prevention in rural areas with high suicide rates, compared with a parallel prevention-as-usual control group, covering a total of 631,133 persons. The effectiveness was also examined in highly populated areas near metropolitan cities (1,319,972 persons). The intervention started in July 2006, and continued for 3.5 years. The primary outcome was the incidence of composite outcome, consisting of completed suicides and suicide attempts requiring admission to an emergency ward for critical care. We compared the rate ratios (RRs) of the outcomes adjusted by sex, age group, region, period and interaction terms. Analyses were performed on an intention-to-treat basis and stratified by sex and age groups.

**Findings:**

In the rural areas, the overall median adherence of the intervention was significantly higher. The RR of the composite outcome in the intervention group decreased 7% compared with that of the control group. Subgroup analyses demonstrated heterogeneous effects among subpopulations: the RR of the composite outcome in the intervention group was significantly lower in males (RR = 0.77, 95% CI 0.59–0.998, p = 0.0485) and the RR of suicide attempts was significantly lower in males (RR = 0.39, 95% CI 0.22–0.68, p = 0.001) and the elderly (RR = 0.35, 95% CI 0.17–0.71, p = 0.004). The intervention had no effect on the RR of the composite outcome in the highly populated areas.

**Interpretation:**

Our findings suggest that this community-based multimodal intervention for suicide prevention could be implemented in rural areas, but not in highly populated areas. The effectiveness of the intervention was shown for males and for the elderly in rural areas.

**Trial Registration:**

ClinicalTrials.gov NCT00737165 UMIN Clinical Trials Registry UMIN000000460

## Introduction

Suicide is a devastating event for individuals, families, and communities. The World Health Organization estimates that nearly 1,000,000 people worldwide die from suicide every year. [1] Several reviews have indicated that multilevel and multimodal interventions would be the strategy of choice for suicide prevention in the community. [2]–[4] A synergistic effect between interventions would be theoretically possible. [4] However, the low rate of suicide in the general population has made it difficult for trials to detect differences between groups with statistical power. Indeed, there are few studies that have reported the effect of interventions on suicide rates. [2]–[4].

We examined the effectiveness of a community-based multimodal intervention for suicide prevention in rural areas where the suicide rate was high, with a non-randomised comparative intervention trial using parallel prevention-as-usual control. The effectiveness was also examined in highly populated areas near metropolitan cities. In the study, a large population size and an appropriate observational period to observe enough suicidal behaviour, and thus to obtain enough statistical power, were used. In addition, preplanned subgroup analyses were performed to detect effects of the intervention in specific subpopulations.

## Methods

### Study Designs

**Figure 1:**
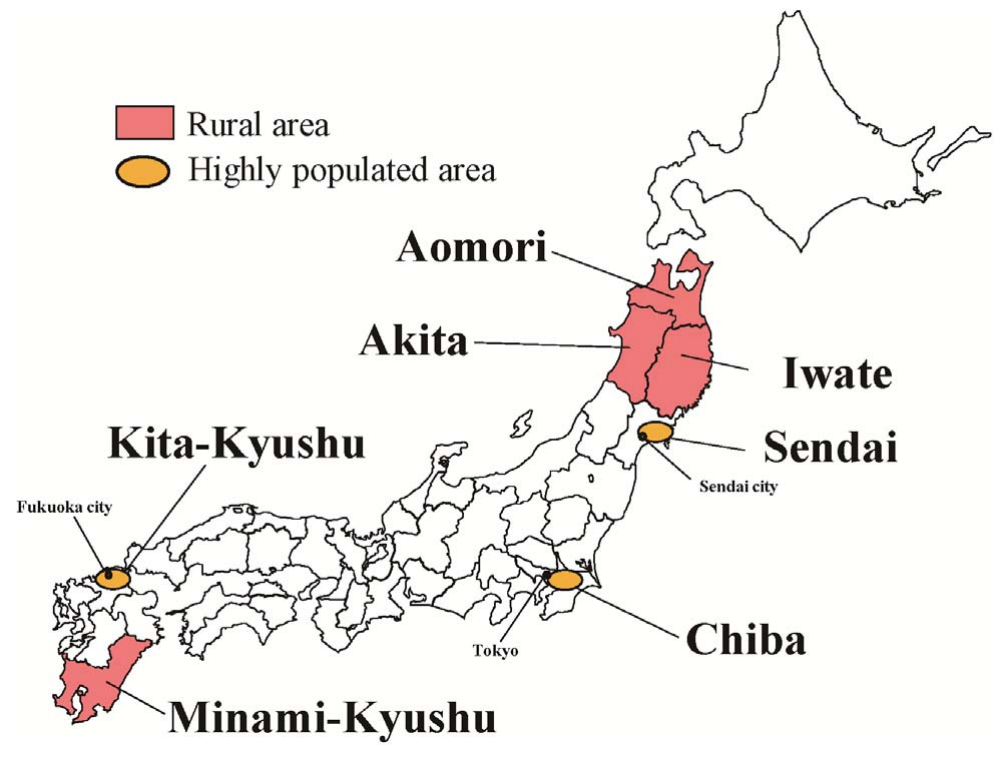
Location map of the study areas. Pink-coloured areas indicate rural study areas. Orange-coloured areas indicate highly populated study areas.

**Figure 2:**
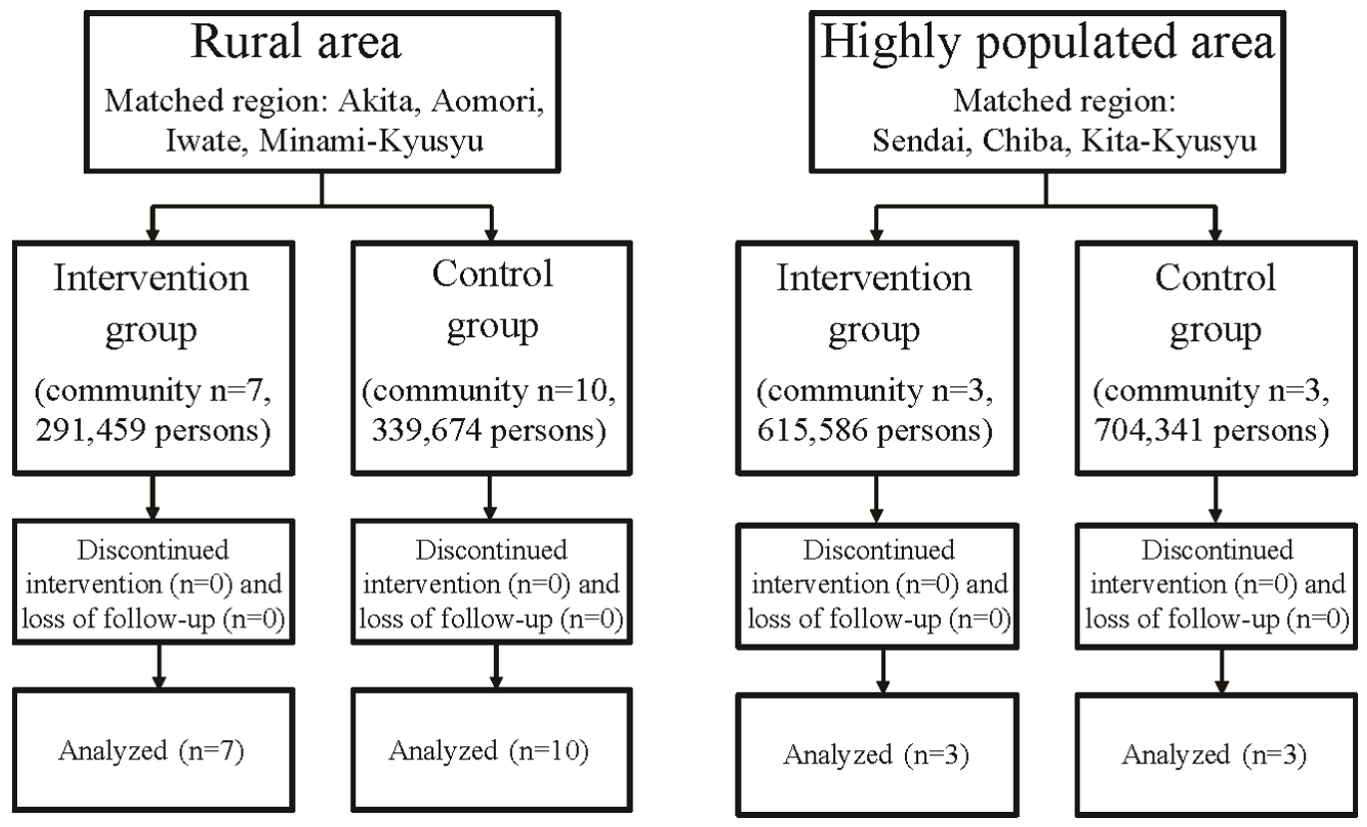
Flow chart of the study.

We set two areas, rural areas and highly populated areas, as the study targets (Figure 1). Rural areas with a high suicide rate were the primary targets, because these were the areas of focus in the previous community interventions in Japan, which are the basis of the interventions in the present study. [5]–[7] The entire population was the target of the intervention. The study matched pairs of intervention groups and control groups with past suicide rates and population size. The participants in the rural areas were the inhabitants living in four matched pairs of intervention groups and control groups (consisting of 17 communities, Figure 2). Highly populated areas near metropolitan cities were another target in this study because the suicide rate in these areas had increased prior to the present study. [8] In highly populated areas, two neighbouring communities were designated as the intervention and control groups, respectively. The participants in the highly populated areas were the inhabitants living in three matched pairs of intervention group and control group (consisting of six communities, Figure 2).

A community-based multimodal intervention for suicide prevention was developed [9] by extending the findings from previous studies focused on depressive elderly living in rural areas of Japan. [5]–[7] The intervention intended to reinforce human relationships and connectedness in the community by focusing on building social support networks within the general public and the health-related resources. The essential components are listed in Table 1. As shown here, the intervention was multilevel and multimodal, targeting the entire population in the participating communities. Leadership involvement was an important factor for the effective implementation of long-term programs by creating society commitment at multiple levels and establishing community support networks. Education and awareness programs aimed to reduce the stigmatisation of mental illness and suicide. The programs also aimed at improving the recognition of suicide risk and facilitating help-seeking and access to mental health services through improved understanding of the causes and risk factors for suicidal behaviour. Training programs targeting gatekeepers and care providers aimed to facilitate their roles in early detection within potentially vulnerable populations and to increase preventive functions. The screening programs aimed to identify at-risk individuals in the community and direct them to treatment. In addition, the program recommended that the local health authorities provide appropriate care for suicide survivors to support their grief work, if necessary.

**Table 1:** 

	Intervention Level	Target	Objectives and Actions
1	Leadership involvement	Local government	Leadership involvement is a key to effectively implementing long-term programs that utilize a commitment of society at multiple levels and succeed in establishing community support networks. Messages from the mayor have a strong impact on the efficiency of community development and community networking.
			a) Publicizing messages from the mayor to all officials and citizens reminding them of the importance of suicide prevention.
			b) Establishing a regional committee dedicated to suicide prevention chaired by the mayor to promote organization-wide awareness of mental health and suicide prevention and facilitate the collaboration of different sections of the local government.
			c) Formalizing the roles of each service section and promoting pathways to build social support networks within the public and health-related resources, intending to reinforce human relationships and connectedness in the community.
2	Education and awareness programs	Public	The education and awareness programs aim to reduce stigmatization of mental illness and suicide and to improve recognition of suicide risk and facilitation of help seeking.
			a) Waging a campaign for general public education (public events, posters, websites, placards, leaflets and brochures with information about help available locally, self-tests, warning signals and treatment options and announcements of regional educational activities like lectures and seminars).
			b) Providing regional educational opportunities like lectures and seminars to improve understanding of the causes and risk factors for suicidal behavior, particularly mental illness. The programs also cover awareness of availability of social resources and referral procedures for people potentially at risk.
3	Gatekeeper training	Community or organizational gatekeepers	Training programs targeting gatekeepers (community leaders, priests, telephone hotlines, social services, youth workers, geriatric care providers, police, physicians, nurses, pharmacists, mental health providers, and those employed in institutional settings, such as schools) aimed to facilitate their playing important roles in early detection within potentially vulnerable populations and increasing preventive functions. These programs also promote organization-wide awareness of mental health and suicide and facilitate access to mental health services.
			a) Training community or organizational gatekeepers to provide them with an opportunity to identify at-risk individuals within different target populations and direct them to appropriate social and/or mental health services.
4	Supporting individuals at high risk	Individuals at high risk	Home visiting and regional social gatherings aim to reinforce human relationships and connectedness in the community. Screening aims to identify at-risk individuals and direct them to treatment.
			a) Home visiting by regional public health nurses and psychiatrists.
			b) Setting up regional social gatherings.
			c) Screening to identify at-risk individuals and direct them to treatment or follow-up care providers. The focus may be on suicidal behavior directly or on risk factors, such as depression or substance abuse.
			d) Support for self-help activities for high-risk groups, i.e., suicide attempters, to facilitate access to professional help.

* The intervention programs focused on building social support networks within the general public and in health-related resources, intending to reinforce human relationships and connectedness in the community.

** A suicide leaves behind more victims than just the individual, as family, friends, co-workers, and the community can be impacted in many different and unique ways following a suicide. In this study, the program recommended that the local government provide appropriate care for suicide survivors (a person who survives a suicide completer; a suicide griever) to support their grief work, if necessary. Support the activities of self-help groups for suicide survivors and other related organizations.

Local governments and the local health authorities collaborated and implemented the intervention programs in accordance with the manual (in Japanese; www.mhlw.go.jp/seisakunitsuite/bunya/hukushi_kaigo/shougaishahukushi/jisatsu/index.html,) developed by the program committee of the NOCOMIT-J group. They implemented the intervention with their own budgets. The participants were not blind to the intervention, which started in July 2006. As short duration interventions did not seem to have any detectable effect [2], the intervention continued for 3.5 years. The intervention in the control group was suicide prevention activities as usual. The list of the programs was opened to the control group when the heads of the local governments agreed to participate in this trial. However, we did not show them the detailed intervention manual. The study monitoring and the data collection were conducted in both the intervention group and control group.

The primary outcome was the incidence of composite outcome, consisting of completed suicides and suicide attempts that required admission to an emergency ward for critical care. Secondary outcomes were as follows: 1) incidence of completed suicides, 2) incidence of suicide attempts that required admission to an emergency ward for critical care, and 3) proportion of adherence with the required components of the intervention described in the manual. For the adherence assessments, information regarding the implementation of the programs described in the manual was collected every 6 months from the month when the study started in all participating regions. The binary questions for the adherence items were prepared based on the essential components listed in Table 1 and were collected from the local health authorities. The reporters of events were not blind to the intervention. Death certificates from the Vital Statistics Records (Ministry of Health, Labour, and Welfare, Japan) for the participating regions from 2003 to 2009 were used every year to collect the following data items: International Classification of Diseases 10th Revision code for intentional self-harm (ICD-10 codes X60–X84), sex, age, and region code. In this study, a suicide attempt patient was defined as a self-harmed individual transported by regional ambulance service and admitted to an emergency ward for critical care. In Japan, fees for ambulance services are covered by the National Health Insurance System, which allows virtually all suicide attempters access to emergency medicine when requested. The following information was collected from the Regional Ambulance Services every 6 months from 2003 to 2009: type of transportation, date of notification, region code, severity, sex and age. Therefore, the data on suicide attempts systematically collected in this study were reliable. The total population numbers by the community, sex and age groups were collected every year from the National Basic Resident Registration System.

### Statistical Analysis

In the primary analysis, we compared the rate ratios (RRs) of incidence of the composite outcome as adjusted by covariates for the effect of the intervention. Marginal models (link function; log, distribution; Poisson) with generalised estimating equations [10] were used to examine the effect of the intervention adjusted by sex, age group (under 25, 25–65, over 65 years), region, period (6-month) and interaction terms (interventions × periods). These variables are well known risk factors from past epidemiological studies and they serve as effect modifications in interventional studies. [11] On the other hand, it takes some time to set up and implement the intervention programs in the community. Therefore, the effects of the intervention would be time-dependent. The 6-month periods were chosen to minimise varying populations. The analysis calculated RRs and their 95% confidence intervals (CI). We conducted an interim analysis 2 years after starting to evaluate the achievement of the primary objective. Therefore, the significance level in the final analysis was set at 0.0492 for the two-sided test based on the method of O’Brien and Fleming. [12].

Sample sizes to be used in the study were calculated based on the assumptions of the suicide rates from 2002 to 2004 in the participating regions. Although the estimated sample sizes were not adjusted for sex, age groups and regions, if all assumptions were met, the statistical power would be over 80%. [9].

Secondary outcomes were analysed to examine whether the rates of completed suicides and suicide attempts were significantly reduced in the intervention group when compared with those of the control group, respectively. Adherence to the interventions was also examined.

Preplanned subgroup analyses of the primary and secondary outcomes by sex and age groups (under 25, 25–65, over 65) were performed. Because these variables were the known risk factors and effect modifications, we also used them for the modelling in the primary analysis. No multiplicity adjustments were made, given the exploratory nature of the analyses.

All analyses were done on an intention-to-treat basis. Statistical analyses were performed using SAS version 9.2 software (SAS Institute Inc., Cary, North Carolina).

The study protocol was approved by the Central Research Ethics Committee of Japan Foundation for Neuroscience and Mental Health. The protocol was also approved by the local Ethics Committees of affiliated universities or institutes in the participating regions. (Aomori region: Kuroishi General Hospital Ethics Committee (http://hospital-kuroishi.jp/) and Hirosaki University Ethics Committee (http://www.hirosaki-u.ac.jp/), Akita region: Akita University Ethics Committee (http://www.akita-u.ac.jp/honbu/), Iwate region: Iwate University Ethics Committee (http://www.iwate-med.ac.jp/), Minami-Kyusyu region: Keio University Ethics Committee (http://www.med.keio.ac.jp/), Sendai region: Tohoku Bunka Gakuen University Ethics Committee (http://www.tbgu.jp/univ/) and Sendai City Hospital Ethics Committee (http://hospital.city.sendai.jp/), Chiba region: National Center Of Neurology And Psychiatry Ethics Committee (http://www.ncnp.go.jp/), Kita-Kyusyu region: University of Occupational And Environmental Health Ethics Committee (http://www.uoeh-u.ac.jp/JP/index.html). We did not directly contact all participants and collect data from all individuals (all data were anonymous) in this study. Therefore, we did not obtain written informed consent from individuals living in the participating regions. Instead, the regional investigators obtained the written authorisation to conduct the study from the head of the local governments on behalf of all inhabitants and announced it as verbal consent in all by public publications in the participated regions. The processes approved the Central Research Ethics Committee and the local Ethics Committees and complied with the Ethical Guidelines for Epidemiology Research (published by the Ministry of Health, Labour, and Welfare, Japan, http://www.niph.go.jp/wadai/ekigakurinri/guidelines.pdf).

The present study is in accordance with the Transparent Reporting of Evaluations with Nonrandomized Designs (TREND) statement [13], [14] and the trial protocol was registered at ClinicalTrials.gov (NCT00737165) and UMIN-CTR (UMIN000000460).

### Role of the Funding Source

The study was conceived and developed by the NOCOMIT-J group, and was funded by the Ministry of Health, Labour, and Welfare of Japan. The Japan Foundation for Neuroscience and Mental Health was the sponsor. Neither the funder nor the sponsor had any role in study design, data collection, data analysis, data interpretation, or writing of the report. YO made the final decision to submit for publication.

## Results

### Demographic Information of the Participating Regions

The trial flow chart is shown in Figure 2. Population characteristics of the participating areas at baseline are shown in Table 2. In the rural study areas, the total population was 631,133 in 2006. In the highly populated study areas, the total population was 1,319,972 in 2006. Numbers of completed suicides, suicide attempts requiring admission to an emergency ward for critical care, and populations from 2003 to 2009 in the rural areas and the highly populated areas are listed in Table 3 and Table 4, respectively.

**Table 2:** Population characteristics at baseline (2006.1–6) in rural and highly populated areas N (%).

	Group 1		Group2	
	(Rural areas)		(Highly populated areas)	
	Intervention	Control	Intervention	Control
	n = 7	n = 10	n = 3	n = 3
All	291,459	339,674	615,586	704,341
Sex				
Male	136,399 (47)	159,380 (47)	310,301 (50)	348,153 (49)
Female	155,060 (53)	180,294 (53)	305,285 (50)	356,188 (51)
Age				
under 25	47,892 (16)	52,867 (16)	103,218 (17)	119,512 (17)
25–64	157,887 (55)	181,153 (53)	407,801 (66)	448,270 (64)
65 and over	85,680 (29)	105,654 (31)	104,567 (17)	136,559 (19)
Region				
Aomori	35,668 (12)	60,695 (18)	–	–
Akita	59,237 (20)	66,678 (20)	–	–
Iwate	55,416 (19)	61,589 (18)	–	–
Minami-Kyushu	141,138 (48)	150,712 (44)	–	–
Sendai	–	–	160,368 (26)	197,915 (28)
Chiba	–	–	411,025 (67)	425,177 (60)
Kita-Kyushu	–	–	44,193 (7)	81,259 (12)

**Table 3:** 

		Intervention							Control						
		Combined		Completedsuicide		Suicideattempt		Population	Combined		Completedsuicide		Suicideattempt		Population
		N	Rate	N	Rate	N	Rate	N	N	Rate	N	Rate	N	Rate	N
Before	2003.1-6	128	86.1	68	45.7	60	40.4	297,397	131	75.3	77	44.2	54	31.0	348,092
	2003. 7-12	91	61.4	68	45.9	23	15.5	296,447	95	54.8	74	42.7	21	12.1	346,639
	2004.1-6	126	85.2	105	71.0	21	14.2	295,655	94	54.4	69	40.0	25	14.5	345,415
	2004. 7-12	70	47.5	49	33.3	21	14.3	294,665	122	71.0	73	42.5	49	28.5	343,825
	2005.1-6	77	52.5	57	38.8	20	13.6	293,589	80	46.7	56	32.7	24	14.0	342,382
	2005. 7-12	102	69.8	51	34.9	51	34.9	292,467	138	81.0	69	40.5	69	40.5	340,927
Reference	2006. 1-6	91	62.4	62	42.5	29	19.9	291,459	139	81.8	76	44.7	63	37.1	339,674
Study	**2006. 7-12**	98	67.6	72	49.6	26	17.9	290,122	89	52.7	57	33.8	32	19.0	337,668
period	**2007. 1-6**	89	61.6	56	38.8	33	22.8	288,882	103	61.3	62	36.9	41	24.4	335,894
	**2007. 6-12**	66	45.9	41	28.5	25	17.4	287,276	103	61.8	57	34.2	46	27.6	333,409
	**2008. 1-6**	73	51.1	49	34.3	24	16.8	285,773	92	55.6	60	36.2	32	19.3	331,133
	**2008. 7-12**	71	49.9	44	30.9	27	19.0	284,379	128	77.8	80	48.6	48	29.2	328,951
	**2009. 1-6**	93	65.7	61	43.1	32	22.6	283,090	90	55.0	61	37.3	29	17.7	326,977
	**2009. 6-12**	72	51.1	54	38.3	18	12.8	281,763	114	70.1	70	43.1	44	27.1	325,146

Combined: Completed suicide and suicide attempt.

Rate: per 10,000 persons, per year.

**Table 4:** 

Highly populated areas															
		Intervention							Control						
		Combined		Completedsuicide		Suicideattempt		Population	Combined		Completedsuicide		Suicideattempt		Population
		N	Rate	N	Rate	N	Rate	N	N	Rate	N	Rate	N	Rate	N
Before	**2003.1-6**	**147**	**48.3**	**71**	**23.3**	**76**	**25.0**	**608,545**	176	50.2	99	28.3	77	22.0	700,674
	**2003. 7-12**	**154**	**50.5**	**74**	**24.3**	**80**	**26.2**	**609,571**	212	60.5	101	28.8	111	31.7	701,360
	**2004.1-6**	**134**	**43.9**	**66**	**21.6**	**68**	**22.3**	**610,644**	168	47.9	79	22.5	89	25.4	702,094
	**2004. 7-12**	**136**	**44.5**	**70**	**22.9**	**66**	**21.6**	**611,912**	171	48.7	84	23.9	87	24.8	702,467
	**2005.1-6**	**160**	**52.2**	**75**	**24.5**	**85**	**27.7**	**613,223**	199	56.6	94	26.7	105	29.9	702,882
	**2005. 7-12**	**122**	**39.7**	**61**	**19.9**	**61**	**19.9**	**614,385**	174	49.5	87	24.7	87	24.7	703,589
Reference	**2006. 1-6**	**166**	**53.9**	**70**	**22.7**	**96**	**31.2**	**615,586**	197	55.9	97	27.5	100	28.4	704,341
Study	**2006. 7-12**	**202**	**65.5**	**80**	**25.9**	**122**	**39.5**	**617,137**	208	59.0	83	23.5	125	35.5	705,159
period	**2007. 1-6**	**164**	**53.0**	**68**	**22.0**	**96**	**31.0**	**618,734**	208	58.9	89	25.2	119	33.7	706,016
	**2007. 6-12**	**154**	**49.6**	**74**	**23.8**	**80**	**25.8**	**620,562**	190	53.7	91	25.7	99	28.0	707,088
	**2008. 1-6**	**148**	**47.6**	**81**	**26.0**	**67**	**21.5**	**622,435**	202	57.0	89	25.1	113	31.9	708,205
	**2008. 7-12**	**165**	**52.9**	**67**	**21.5**	**95**	**30.4**	**624,319**	222	62.6	87	24.5	135	38.0	709,661
	**2009. 1-6**	**156**	**49.8**	**80**	**25.5**	**76**	**24.3**	**626,250**	190	53.4	86	24.2	104	29.2	711,167
	**2009. 6-12**	**137**	**43.7**	**51**	**16.3**	**86**	**27.4**	**626,963**	208	58.4	92	25.8	116	32.6	711,837

Combined: Completed suicide and suicide attempt.

Rate: per 10000 persons, per year.

**Figure 3:**
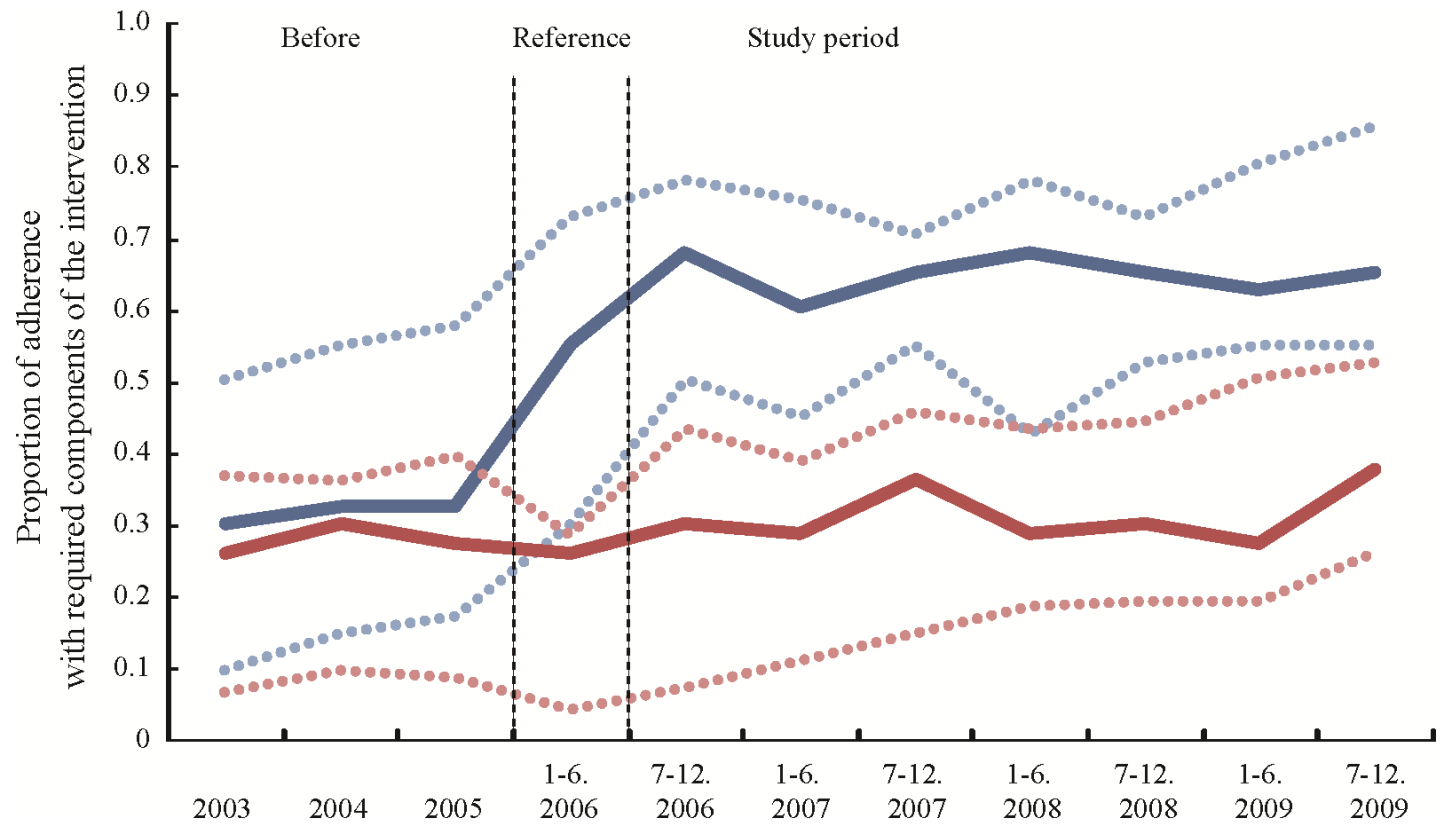
Figure 3 shows the proportion of adherence with required components of the intervention in the rural areas. The blue line indicates the proportion of the intervention group, and the red line indicates that of the control group. The dotted lines indicate interquartile ranges. The proportion is shown from the 3.5 years before the start of the study period. The six-month period before the start of the study period was the reference period.

**Figure 4:**
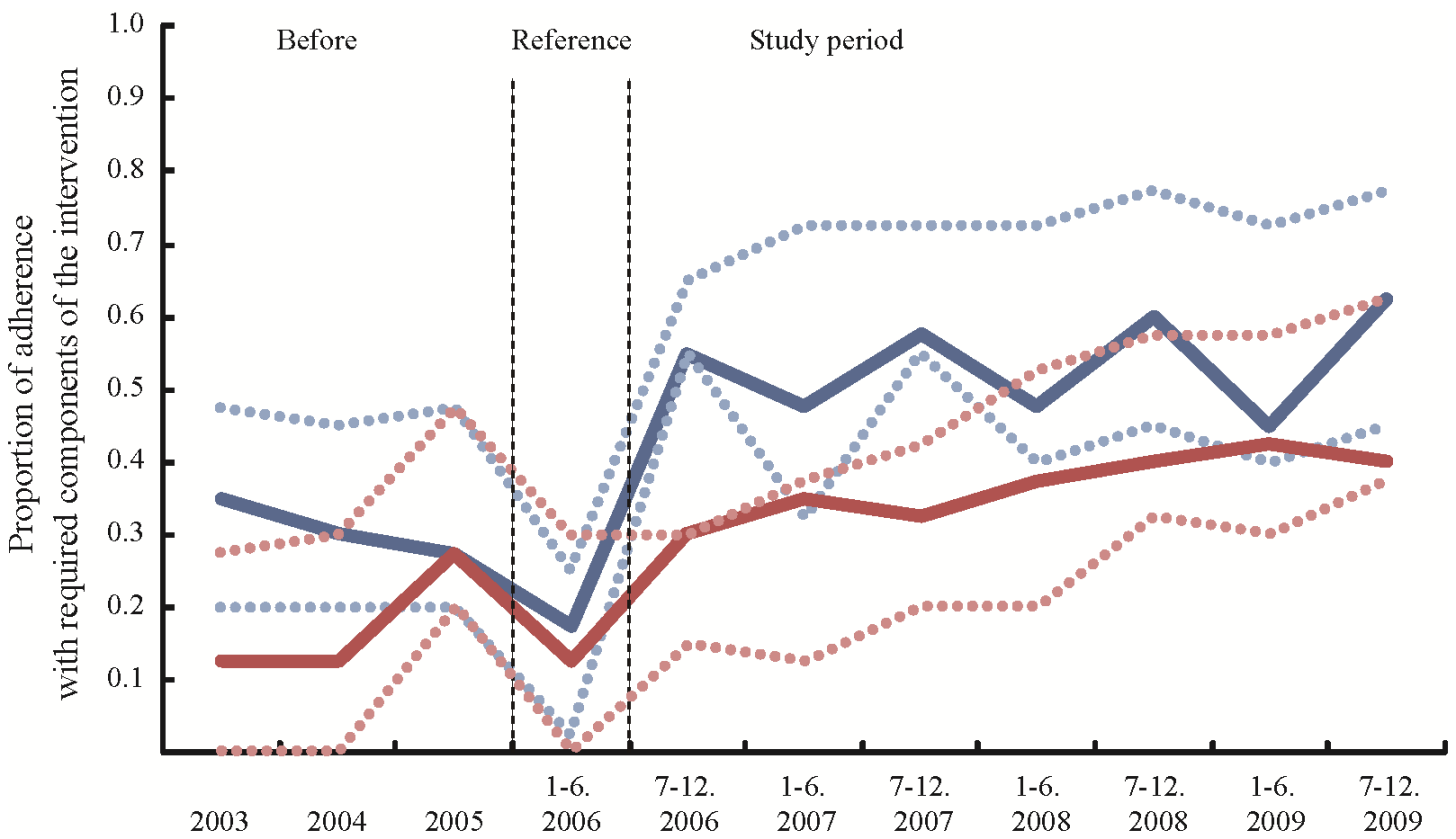
Figure 4 shows the proportion of adherence with required components of the intervention in the highly populated areas. The blue line indicates the proportion of the intervention group, and the red line indicates that of the control group. The dotted lines indicate interquartile ranges. The proportion is shown from the 3.5 years before the start of the study period. The six-month period before the start of the study period was the reference period.

The adherence to the suicide prevention programs implemented in the participating areas is shown in Figure 3 and 4. In rural areas, the overall median adherence of the intervention group was 0.65 and significantly higher than that obtained from the control group (beta = 0.42, 95% CI 0.12–0.72, p = 0.0056). On the other hand, in highly populated areas, the overall median adherence of the intervention group was 0.55, not different from that of the control group (beta = 0.35, 95% CI −0.01–0.71, p = 0.0552).

The interim analysis conducted 2 years after the start of the intervention demonstrated that the incidence rates of the composite outcome were similar between the intervention group and control group in the rural areas (RR = 0.99, 95% CI 0.79–1.06, p = 0.257). As the result, the study was continued until the end of the planned period.

**Figure 5:**
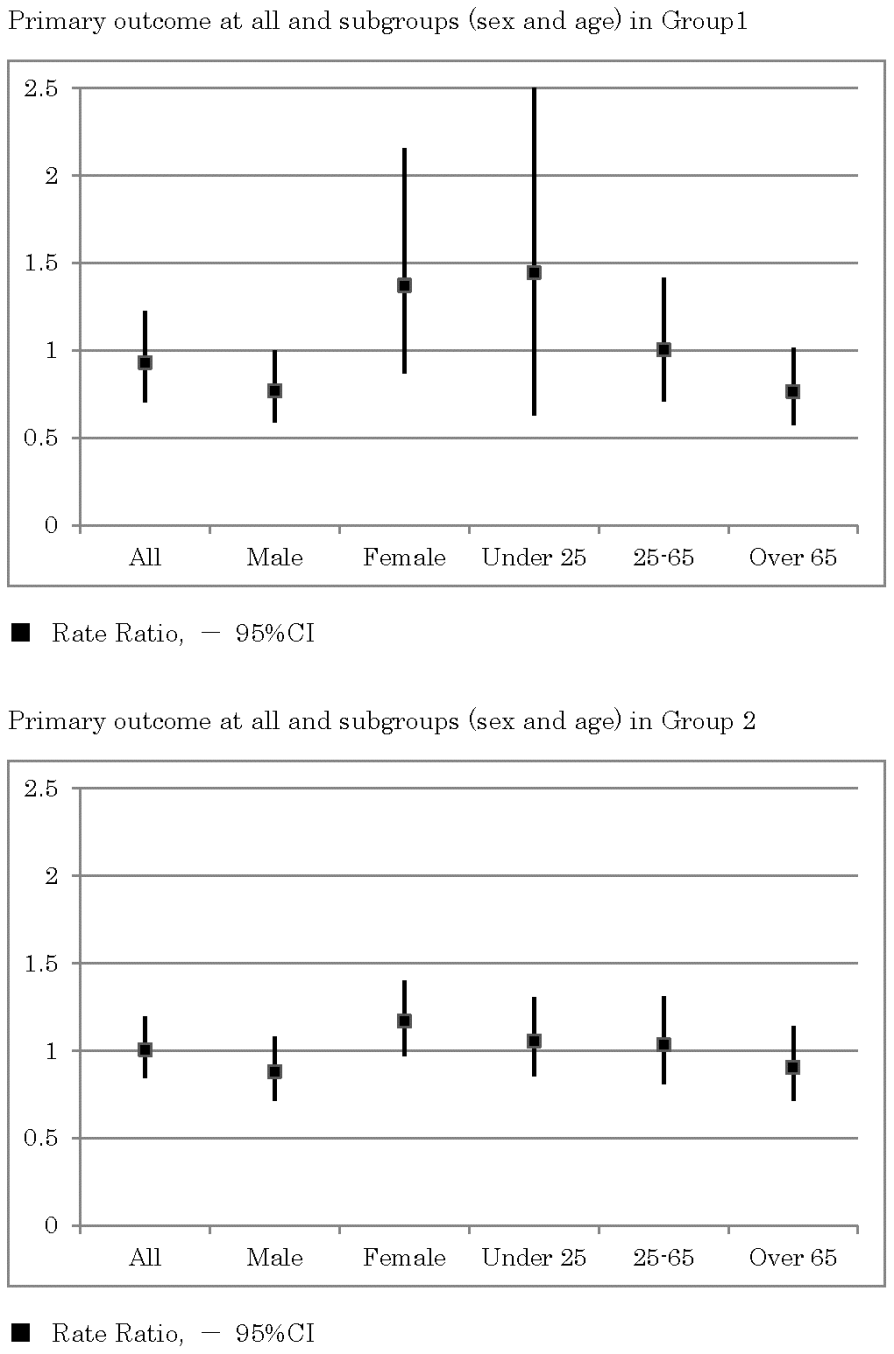
Primary outcome (composite outcome, consisting of completed suicides and suicide attempts requiring admission to an emergency ward for critical care) for all and for subgroups (sex and age) in rural areas and in highly populated areas.

As shown in Figure 5, in the rural areas the incidence rates of the composite outcome in the intervention group were slightly lower than those obtained from the control group (RR = 0.93, 95% CI 0.71–1.22, p = 0.598). A subgroup analysis demonstrated that the incidence rates in the intervention group were significantly lower in males (RR = 0.77, 95% CI 0.59–0.998, p = 0.0485). Also, the analysis demonstrated that the incidence rates in the intervention group were lower in the elderly over 65 years old (RR = 0.76, 95% CI 0.57–1.01, p = 0.062), while the incidence rates were higher in females (RR = 1.34, 95% CI 0.87–2.15, p = 0.174) and in participants younger than 25 years old (RR = 1.44, 95% CI 0.63–3.31, p = 0.386).

In the highly populated areas, the incidence rates of the composite outcome were similar between the intervention group and control group (RR = 1.00, 95% CI 0.85–1.19, p = 0.961).

**Figure 6:**
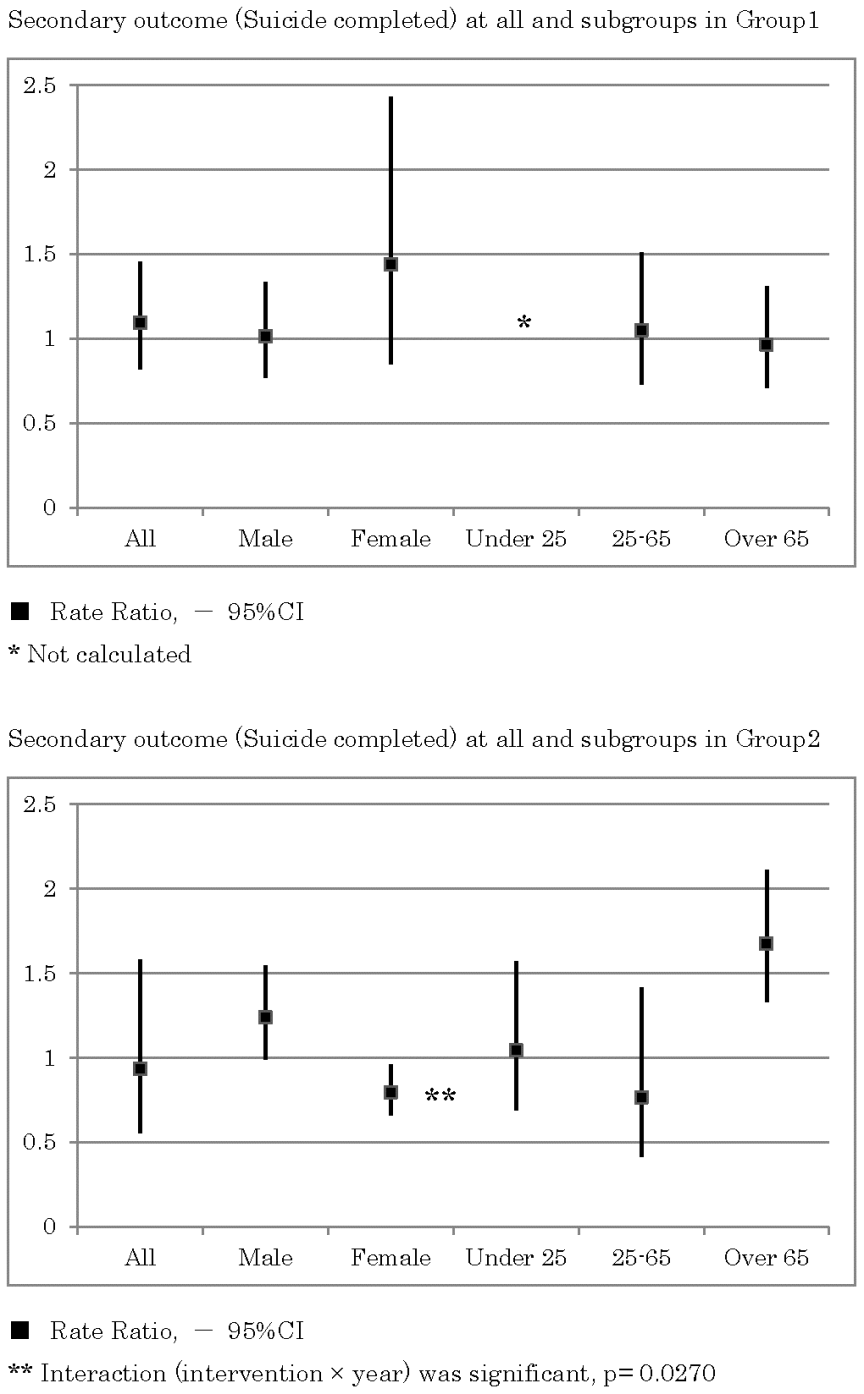
Secondary outcome (completed suicides) for all and for subgroups in rural areas and in highly populated areas.

As shown in Figure 6, in the rural areas the incidence rates of completed suicides were similar between the intervention group and control group (RR = 1.09, 95% CI 0.82–1.45, p = 0.550). A subgroup analysis demonstrated that the incidence rates in the intervention group were higher in females (RR = 1.44, 95% CI 0.85–2.43, p = 0.177).

**Figure 7:**
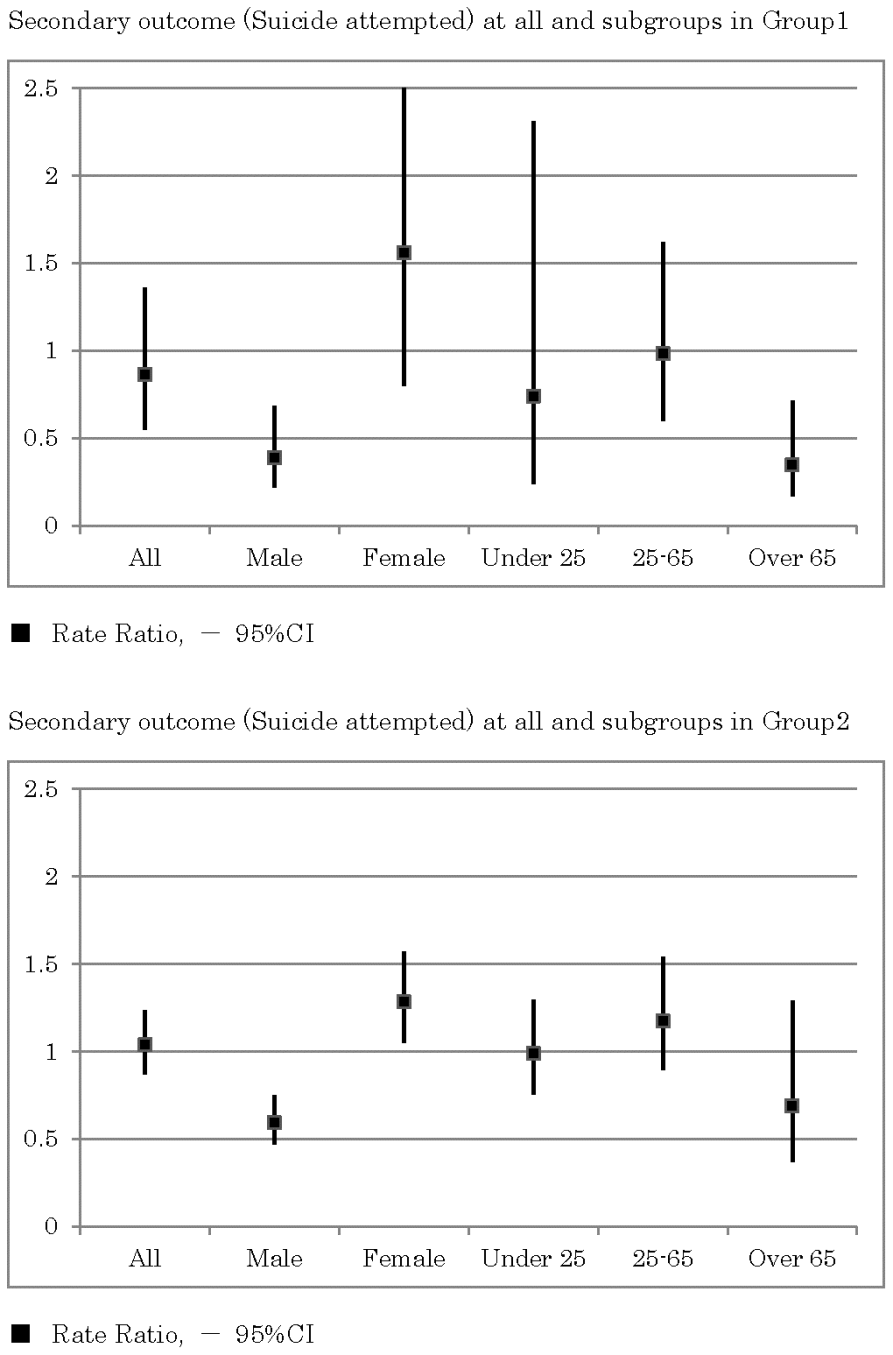
Secondary outcome (suicide attempts) for all and for subgroups in rural areas and in highly populated areas.

As shown in Figure 7, in the rural areas, the incidence rates of suicide attempts requiring admission to an emergency ward for critical care in the intervention group were slightly lower than those obtained from the control group (RR = 0.86, 95% CI 0.55–1.36, p = 0.524). A subgroup analysis demonstrated that the incidence rates in the intervention group were significantly lower in males (RR = 0.39, 95% CI 0.22–0.68, p = 0.001) and the elderly over 65 years old (RR = 0.35, 95% CI 0.17–0.71, p = 0.004). The subgroup analysis demonstrated that the incidence rates in the intervention group were lower in participants younger than 25 years old (RR = 0.74, 95% CI 0.24–2.31, p = 0.605), while the incidence rates were higher in females (RR = 1.56, 95% CI 0.80–3.04, p = 0.193).

As shown in Figure 6 and 7, in the highly populated areas, the RRs of suicide attempts and completed suicide demonstrated heterogeneous effects.

## Discussion

In the present study, the intervention had unclear effects on the overall rate ratio of the composite outcome in rural areas where the suicide rate was high. The overall rate ratio of the composite outcome in the intervention group was 7% lower than that obtained from the control group. This was possibly because the intervention had some heterogeneous effects on different subpopulations. Indeed, the RR in males was significantly lower. It was also demonstrated that the RR in the elderly was lower, while it was higher in females and in younger participants. Interestingly, our secondary analysis suggested that the effects were more apparent in the incidence of suicide attempts than that of completed suicides. It was demonstrated that the RR of suicide attempts in the intervention group was significantly lower in males and in the elderly. Thus, this study apparently demonstrated the heterogeneous effects of the intervention on the different subpopulations.

The beneficial effect of the intervention in males is consistent with a previous report showing a significant reduction of suicide rate through a community-based intervention in the US Air Force, in which about 84% of participants were males.^15^ Interventions used in the study aimed to establish a seamless system of services across multidisciplinary human services with very strong and vertical leadership involvement. From another point of view, it is well known that about 90% of suicides are associated with mental illness, i.e., depression and other affective disorders, schizophrenia, substance/alcohol-related disorders and personality disorders. [16], [17] The interventions in the US Air Force study covered not only depression but also a range of psychosocial risk factors for suicide, and thus were multilevel and multimodal. Although the sample size was quite large, the US Air Force study was a quasi-experimental pre-post design conducted in a single organisation (Table 5). In the present study, we conducted a parallel comparative trial in multiple regions to examine the effectiveness of a similar approach. Therefore, our study has reproduced and extended the findings of the US Air Force study. Here, it is concluded that a community-based multimodal intervention would be recommended for males. It is still unclear which component of the complex intervention programs is especially important for males.

**Table 5:** Related studies.

Study	Population	Study Size, Sites	Sex, Age	Study Design	Intervention	Pre suicide rate	Duration	Compliance	Outcome	Analysis	Results
Knox et al., BMJ. 2003 Dec 13; 327(7428):1376.	US Air Force personnel	5,260,292	About 84% men	(Quasi-experimental) pre-post design	Multimodal (10 initiatives)	1990–6 (median 13.1)	5 years	Over 80%	Completed suicides, homicide, accidental death,?family?violence	?2 test for linear trend with the Mantel-Haenszel, and relative-risk (RR),(No adjustment for sex and age)	Significant 33% reduction of suicide (RR 0.67, 0.57–0.80) compared to control
Hegerl et al., Psychol Med. 2006; 36(9):1225–33.	Inhabitants living in the city	720,000	No data (no differences between pre-post)	(Non-randomised) concurrent comparative (a city vs. a city) design	4 levels; Training of primary care physicians, public campaign for depression, corporation with facilitators and self-help activities support	Intervention (about 18) vs. control (about 15)	2 years	Unknown percentage (details of activities only)	Completed suicide, suicide attempted, And combined (suicide acts)	Change rate and χ2 test (stratified sex and years, but not adjusted)	19.4% to 24% reduction suicide acts rate (p = 0.082, 0.004) compared to control
		Intervention: Nuremberg, 480,000									
		Control: Wurzburg 270,000									
Szanto et al., Arch Gen Psychiatry. 2007; 64(8): 914–20.	A region with a igh suicide rate in Hungary	127,000	48% were men, 22% were over age 60	(Non-randomised) concurrent comparative(a region vs. a region) design	Training of primary care physicians and nurse, plus telephone psychiatrist consultation	Intervention (median 57.5) vs. control (median 56)	5 years	About 60% (39–90%)	Completed suicides (from police), prescription of anti-depressants, alcohol related death and unemployment	Poisson log-link function, Mixed linear models with repeated measures (adjusted years, stratified sex, but not adjusted age)	No significant difference between intervention and control overall, but female suicide decreased by 34% in intervention and increased by 90% in control; significant decrease compared to county and country (Hungary) levels.
		Intervention: Kiskunhalas,73,000 (44,000 in villages and 29,000 in a town) with 28 GPs									
		Control: Bacs-Kuskun,54,000 (22,000 in villages and 32,000 in a town)									
NOCOMIT-J	Inhabitants living in high suicide-rate areas in Japan	631,133 (rural area)	47% were men, 30% were 65 over aged	(Non-randomised) controlled (matched) concurrent comparative (2 areas and 4 regions) design	Multimodal (4 levels)	Intervention (median 42.5)vs. control (median 42.5) in rural areas	3.5 years	About 70% in rural areas, About 55% in highly populated areas	Completed suicides from government), suicide attempts, and combined	Poisson log-link function, Marginal models in repeated measures with GEE (adjusted sex, age and years)	9% reduction, not significant, but men 23% (p = 0.0485), over 65 24%(p = 0.062) reduction compared to concurrent control
		7 intervention vs. 10 control									
		1,319,927 (highly populated area)				Intervention (median 22.9) vs. control (median 26.7) in highly populated areas					
		4 interventions vs. 4 controls									

In this study, we developed our intervention by extending the findings from previous studies, focusing especially on elderly. [5]–[7] As we expected, beneficial effects of the intervention in the elderly were consistent with these previous observations. [5]–[7] In their interventions, psychiatrists and public health nurses tried to reinforce human relationships and connectedness in their community by home visiting and regional social gatherings. However, these previous studies were retrospective pre-post designs with one or a few communities. Therefore, our study confirmed the findings for elderly and concluded that the effect would be significant in this group.

As shown in Table 5, there are some reports of multilevel interventions focusing on depression care. [18], [19] These interventions aimed to introduce untreated depressed patients to appropriate care by public awareness campaigns, educating general practitioners and supporting them with mental health professionals, and encouraging collaborative care. A German study, the Nuremberg Alliance Against Depression, reported a reduction in nonfatal suicide attempts in an intervention region compared with a control region but no difference in effect on the rate of completed suicides. [18] In the report from Hungary, the rate of completed suicides in the intervention region was not different from that in the control region. [19] Although the type of our intervention was different from these two studies, the absence of the effect on the rate of completed suicides in the intervention region was also observed in our NOCOMIT-J. On the contrary, as described above, the US Air Force study, which covered various risk factors, showed a significant reduction of the rate of completed suicides. [15] A high rate of adherence to the intervention (over 80%) might help to achieve the objective of lowering completed suicide rates (Table 5).

The incidences of suicidal behaviour were similar between the intervention group and control group in the highly populated areas near metropolitan cities, not unexpected as adherence of the intervention group to the suicide prevention programs was not different from that of the control group. In addition, adherence of the intervention group in highly populated area was relatively low, suggesting the difficulties of implementing the intervention in these areas. Further improvement of the intervention programs would be necessary to make the intervention feasible in the highly populated areas.

There are several limitations of the present study. 1) The study was not a randomised trial. Therefore, we used a matched pair design and a model adjusted for possible confounding factors in the analysis. However, some unmeasured and residual confounders may still persist. We need to perform randomised trials confirming our insights. 2) The study participants, investigators and the reporters of events were not blind to the intervention. Although the outcomes were systematically collected from official records, the study might have some misclassification bias. 3) Adherence to the intervention was limited. The adherence would be improved by investing sufficient budgets and resources.

In conclusion, our findings suggest that the community-based multimodal intervention for suicide could be implemented in the all areas. However, the effectiveness of the intervention are shown for males and for the elderly in rural area. Therefore, it would be recommended for males and for the elderly in rural areas.

## References

[1] World Health Organization (2012) Public health action for the prevention of suicide: A framework. Geneva, Switzerland: WHO Press.

[2] Fountoulakis KN, Gonda X, Rihmer Z (2011) Suicide prevention programs through community intervention. J Affect Disord 130(1–2): 10–6. 20599277 10.1016/j.jad.2010.06.009

[3] Mann JJ, Apter A, Bertolote J, Beautrais A, Currier D, et al (2005) Suicide prevention strategies: a systematic review. JAMA 294(16): 2064–74. 16249421 10.1001/jama.294.16.2064

[4] van der Feltz-Cornelis CM, Sarchiapone M, Postuvan V, Volker D, Roskar S, et al (2011) Best practice elements of multilevel suicide prevention strategies: a review of systematic reviews. Crisis 32(6): 319–33. 21945840 10.1027/0227-5910/a000109 PMC3306243

[5] Ono Y (2004) Suicide prevention program for the elderly: the experience in Japan. Keio J Med 53(1): 1–6. 15096721 10.2302/kjm.53.1

[6] Oyama H, Watanabe N, Ono Y, Sakashita T, Takenoshita Y, et al (2005) Community-based suicide prevention through group activity for the elderly successfully reduced the high suicide rate for females. Psychiatry Clin Neurosci 59(3): 337–44. 15896228 10.1111/j.1440-1819.2005.01379.x

[7] Oyama H, Sakashita T, Ono Y, Goto M, Fujita M, et al (2008) Effect of community-based intervention using depression screening on elderly suicide risk: a meta-analysis of the evidence from Japan. Community Ment Health J 44(5): 311–20. 18363103 10.1007/s10597-008-9132-0

[8] Fujita T (2003) Rapid Increases of Suicide Deaths in Metropolitan Areas. J Natl Inst Public Health 52: 295–301.

[9] Ono Y, Awata S, Iida H, Ishida Y, Ishizuka N, et al (2008) A community intervention trial of multimodal suicide prevention program in Japan: a novel multimodal community intervention program to prevent suicide and suicide attempt in Japan, NOCOMIT-J. BMC Public Health 8: 315. 18793423 10.1186/1471-2458-8-315 PMC2551615

[10] Fitzmaurice GM, Laird NM, Ware JH (2011) Applied Longitudinal Analysis, 2^nd^ Edition. New Jersey: John Wiley & Sons, Inc.

[11] Wasserman D, Wasserman C, editors (2009) Oxford textbook of suicidology and suicide prevention: a global perspective. New York: Oxford University Press Inc.

[12] O’Brien PC, Fleming TR (1979) A multiple testing procedure for clinical trials. Biometrics 35(3): 549–56. 497341

[13] Des Jarlais DC, Lyles C, Crepaz N (2004) Improving the reporting quality of nonrandomized evaluations of behavioral and public health interventions: the TREND statement. Am J Public Health 94(3): 361–6. 14998794 10.2105/ajph.94.3.361 PMC1448256

[14] Armstrong R, Waters E, Moore L, Riggs E, Cuervo LG, et al (2008) Improving the reporting of public health intervention research: advancing TREND and CONSORT. J Public Health (Oxf) 30(1): 103–9. 18204086 10.1093/pubmed/fdm082

[15] Knox KL, Litts DA, Talcott GW, Feig JC, Caine ED (2003) Risk of suicide and related adverse outcomes after exposure to a suicide prevention programme in the US Air Force: cohort study. BMJ 327(7428): 1376. 14670880 10.1136/bmj.327.7428.1376 PMC292986

[16] Arsenault-Lapierre G, Kim C, Turecki G (2004) Psychiatric diagnoses in 3275 suicides: a meta-analysis. BMC Psychiatry 4: 37. 15527502 10.1186/1471-244X-4-37 PMC534107

[17] Cavanagh JT, Carson AJ, Sharpe M, Lawrie SM (2003) Psychological autopsy studies of suicide: a systematic review. Psychol Med 33(3): 395–405. 12701661 10.1017/s0033291702006943

[18] Hegerl U, Althaus D, Schmidtke A, Niklewski G (2006) The alliance against depression: 2-year evaluation of a community-based intervention to reduce suicidality. Psychol Med 36(9): 1225–33. 16707028 10.1017/S003329170600780X

[19] Szanto K, Kalmar S, Hendin H, Rihmer Z, Mann JJ (2007) A suicide prevention program in a region with a very high suicide rate. Arch Gen Psychiatry 64(8): 914–20. 17679636 10.1001/archpsyc.64.8.914

